# A Narrative Review of Self-Reported Scales to Evaluate Depression and Anxiety Symptoms in Adult Obstructive Sleep Apnea Patients

**DOI:** 10.3390/medicina60020261

**Published:** 2024-02-02

**Authors:** Diana Raluca Velescu, Monica Steluta Marc, Daniel Traila, Camelia Corina Pescaru, Patricia Hogea, Noemi Suppini, Alexandru Florian Crisan, Norbert Wellmann, Cristian Oancea

**Affiliations:** 1 Center for Research and Innovation in Precision Medicine of Respiratory Diseases, “Victor Babes” University of Medicine and Pharmacy Timisoara, Eftimie Murgu Square 2, 300041 Timisoara, Romania; velescu.diana@umft.ro (D.R.V.); traila.daniel@umft.ro (D.T.); pescaru.camelia@umft.ro (C.C.P.); hogea.patricia@umft.ro (P.H.); noemi.suppini@umft.ro (N.S.); crisan@umft.ro (A.F.C.); oancea@umft.ro (C.O.); 2 Department of Infectious Diseases, Discipline of Pulmonology, “Victor Babes” University of Medicine and Pharmacy Timisoara, Eftimie Murgu Square 2, 300041 Timisoara, Romania; 3 Research Center for the Assessment of Human Motion, Functionality and Disability (CEMFD), “Victor Babes” University of Medicine and Pharmacy Timisoara, Eftimie Murgu Square 2, 300041 Timisoara, Romania; 4 Doctoral School, “Victor Babes” University of Medicine and Pharmacy Timisoara, Eftimie Murgu Square 2, 300041 Timisoara, Romania; norbert.wellmann@umft.ro

**Keywords:** depression, anxiety, obstructive sleep apnea, self-reported scales

## Abstract

*Background and Objectives*: Obstructive sleep apnea (OSA) is a prevalent chronic condition that has been associated with mental disorders like depression and anxiety. This study intends to provide a practical overview of the most relevant self-reported and self-rating scales that assess depression and anxiety in OSA patients. *Materials and Methods*: A search for articles was performed using PubMed, Google Scholar, and Semantic Scholar using a combination of words for obstructive sleep apnea, depression, anxiety, and scales. The tools were ordered by type (screening and rating) and arranged chronologically according to the year of publication. *Results*: Three scales were identified for assessing depression, which were the Center for Epidemiologic Studies Depression Scale (CES-D), the Hospital Anxiety and Depression Scale (HADS-D), and the Patient Health Questionnaire-9 (PHQ-9). For rating depression, two scales were discussed: the Zung Self-Rating Depression Scale (SDS) and the Beck Depression Inventory (BDI), which has three versions (the BDI, the BDI-II, and the Fast Screen (BDI-FS)). For assessing anxiety, the Generalized Anxiety Disorder-7 (GAD-7) scale was identified. Two scales were reviewed for rating anxiety: the State-Trait Anxiety Inventory (STAI) and the Beck Anxiety Inventory (BAI). Each scale is accompanied by a brief description of its practicality and psychometric qualities and an analysis of its strengths and limitations. *Conclusions*: The findings of this review will contribute to the understanding of the importance of assessing mental health comorbidities in the context of OSA, ultimately guiding clinical practice and future research in this area.

## 1. Introduction

Obstructive sleep apnea is the most common and clinically important sleep-related breathing disorder, with a prevalence ranging from 9% to 38% in the general population [1,2]. It is characterized by repetitive episodes of partial or complete cessation of airflow in the upper airway during sleep that lead to intermittent hypoxia, sleep fragmentation, and impaired oxygen supply to vital organs [3], and it has significant associations with health problems such as cardiovascular disease [4,5,6], metabolic alterations and type 2 diabetes [7,8], cognitive impairment and decline [9], and mental disorders like depression and anxiety [10]. Moreover, patients with OSA manifest excessive daytime sleepiness, which causes car accidents and increases the risk of injury [11,12].

The association between OSA and mental health disorders has gained significant attention recently. Depression and anxiety are highly prevalent in OSA patients, and their presence can exacerbate sleep disturbances and decrease treatment adherence. A recent meta-analysis and systematic review estimated that the prevalences of depressive and anxiety symptoms in a study population with OSA were as high as 35% and 32%, respectively [10]. Moreover, Kaufman et al. showed that OSA patients were 3.11 times more likely to be depressed, 3.68 times more likely to experience anxiety, 2.75 times more likely to have suicidal thoughts, and 2.88 times more likely to experience severe mental stress compared to healthy people [13].

Researchers recognize the presence of psychological symptoms in OSA patients, but the underlying mechanism is still unclear. Studies suggest that the connections between OSA and depression and anxiety symptoms are bidirectional [10,14,15,16,17,18].

Scales are patient-centered tools to assess symptoms that are not directly observable during a clinical examination. These questionnaires may incorporate subjective aspects, reflecting a patient’s personal view and evaluation of a symptom and its effects on their daily life. Therefore, these outcomes are seen as patient-related and offer the benefit of redirecting attention toward the patient, which is necessary in the present biopsychosocial framework of patient-centered care.

This narrative review aims to provide clinicians and researchers with an overview of the scales that are currently available to screen for depression and anxiety and measure their severity in OSA patients. The scales are organized by type (screening or rating) and chronologically by year of publication. After a description and practical application of each scale, its psychometric properties are discussed, along with the instrument’s strengths and limitations.

## 2. Methods

### 2.1. Search Strategy

A comprehensive search strategy was employed to identify relevant studies. Electronic databases, such as PubMed, Google Scholar, and Semantic Scholar, were searched using keywords and Medical Subject Headings (MeSHs) related to “obstructive sleep apnea and depression”, “obstructive sleep apnea and anxiety”, “self-reported scales for depression”, “questionnaires for depression”, “self-reported scales for anxiety”, or “questionnaires for anxiety” and terms associated with “OSA therapy and depression” and “OSA therapy and anxiety”. We also performed a manual search of reference lists from relevant review articles. The search was limited to articles published in English from January 2000 to January 2023.

### 2.2. Inclusion and Exclusion Criteria

Studies were selected based on predetermined inclusion and exclusion criteria. The inclusion criteria were as follows: (1) the study involved human adults over 18 years of age, (2) the diagnosis of OSA was confirmed when a polysomnography or polygraphy recording indicated an Apnea–Hypopnea Index (AHI) of >5 per hour of sleep [19], (3) the study examined identifying depression and anxiety in individuals with obstructive sleep apnea, and (4) the study utilized self-reported scales and questionnaires as assessment tools. Studies were excluded if they (1) referred to pediatric individuals; (2) did not focus on obstructive sleep apnea or mental health disorders; (3) did not utilize self-reported scales and questionnaires as assessment tools; or (4) were conference abstracts, editorials, or case reports without original data.

### 2.3. Study Selection

**Figure 1:**
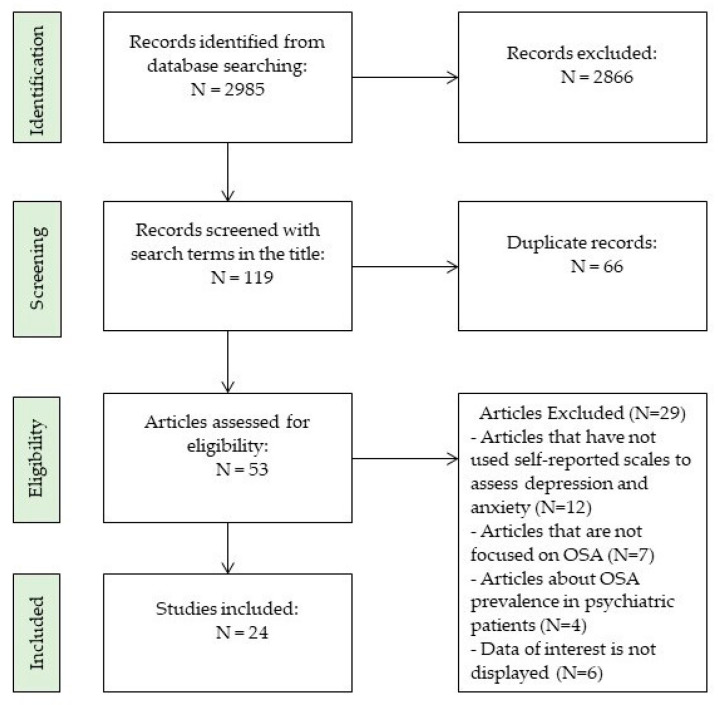
Study selection for narrative review.

Two authors (Diana R. Velescu and Monica S. Marc) screened the titles and abstracts of the identified studies based on the inclusion and exclusion criteria. In addition, full-text articles of potentially relevant studies were retrieved and further assessed for eligibility. Finally, their consensus was reviewed by the other authors to obtain a final agreement on the most relevant scales (Figure 1).

## 3. Results

### 3.1. Depression, Anxiety, and Obstructive Sleep Apnea

#### 3.1.1. Prevalence and Risk Factors

According to the World Health Organization, approximately 280 million people worldwide have depression, and over 300 million people worldwide manifest anxiety. It is estimated that 3.8% of the population experiences depression, including 5% of adults (4% of men and 6% of women) and 5.7% of adults older than 60 [20]. The prevalence of depression in OSA ranges from 5% to 63% in various studies [10,14,21] compared to the general population. These diseases are associated with increased morbidity and mortality and decreased quality of life. The relationship is complex and multifactorial. While the exact mechanisms are not fully understood, several factors contribute to the association between depression, anxiety, and OSA: (1) Sleep disruption. OSA is characterized by disruptions of breathing and frequent awakenings that result in fragmented and low-quality sleep, leading to sleep deprivation and disturbances of sleep architecture. Sleep deprivation and poor sleep quality contribute to mood disturbances, including depressive and anxiety symptoms [22]. (2) Neurotransmitter imbalance. Neurotransmitters like serotonin, norepinephrine, and γ-aminobutyric acid (GABA) play a role in the sleep/wake cycle and mood regulation [23,24]. Neuroimaging research shows that patients with OSA and depression might share some common structural brain abnormalities, like significant reductions in gray matter, including in the hippocampus, anterior cingulate cortex, amygdala, and frontal cortex [25]. (3) Chronic hypoxia, oxidative stress, and inflammation. During episodes of apnea in OSA, oxygen levels in the blood can decrease, leading to intermittent hypoxia. Chronic intermittent hypoxia and oxidative stress resulting from OSA can negatively affect the brain, potentially affecting mood regulation [22,25]. Moreover, OSA patients have shown an inadequate immune response and abnormal activation of the inflammatory response system, increasing the release of pro-inflammatory cytokines. According to a meta-analysis focusing on the relationship between OSA and inflammation, the most prominent inflammatory factors were interleukine-1 (IL-1), interleukine-6 (IL-6), and C-Reactive Protein (CRP) [26]. Bozic et al. found that TNF-α, IL-6, and high-sensitive CRP levels were significantly higher in newly diagnosed OSA patients compared to a healthy control group [27]. A similar immune response is seen in patients with depression, and these pro-inflammatory cytokines may share a possible path between these two conditions [28]. (4) Shared risk factors. Depression, anxiety, and OSA share common risk factors such as obesity, a sedentary lifestyle, and certain medical conditions like cardiovascular disease and diabetes [29,30].

#### 3.1.2. Assessment of Depression and Anxiety

The assessment of depression and anxiety in individuals with OSA involves various methods, including clinical interviews, self-report questionnaires, and diagnostic criteria from standardized classification systems such as the Diagnostic and Statistical Manual of Mental Disorders (DMS-IV and DSM-5) [31,32]. Self-assessment scales offer a standardized, efficient, and cost-effective method for identifying possible cases of depression and anxiety in OSA patients, and they do not require time-consuming training programs (Table 1).

#### 3.1.3. Scales for Depression and Anxiety in OSA Patients

- Center for Epidemiologic Studies Depression Scale (CES-D)

Description: The 20 items in this assessment evaluate the individual’s perceived mood and functioning level over the previous week. These items include four factors, namely depressed affect, positive affect, somatic problems, psychomotor impairment, and interpersonal relationship problems, with a particular focus on depressed affect [33,42]. The original version with 20 items has been reduced to 10 items for the older population [43].

Practical application: The CES-D is available in the original article [33] and online at http://www.chcr.brown.edu/pcoc/cesdscale.pdf. Using the CES-D scale does not incur any financial expenses, as it is available in the public domain. It can be self-administered using a pen or pencil and takes 10 min to complete. Responses use 4-point scales (0—rarely or none of the time (less than 1 day), 1—some or a little of the time (1–2 days), 2—occasionally or a moderate amount of time (3–4 days), and 3—most or all of the time (5–7 days)), with a total score ranging from 0 to 60. A higher score indicates more significant symptoms of depression, weighted by frequency of occurrence in the past week. The recommended cut-off for clinical depression is a score ≥ 16, but better indicators were reported for 20 points [44].

Psychometric properties: This scale has high internal consistency, with α coefficient ranging from 0.81 to 0.92 [45]. A cut-off ≥ 16 shows a sensitivity of 0.87 (95% CI: 0.82–0.92) for cases and a specificity of 0.70 (95% CI: 0.65–0.75). The diagnostic odds ratio (DOR) at this cut-off is 16.2, with a 95% CI of 10.49 to 25.10. However, for a cut-off of 20, a better balance between sensitivity and specificity has been observed, with values of 0.83 for sensitivity and 0.78 for specificity. The DOR at this threshold is 16.64 [44].

Strengths and limitations: This scale has been effectively validated and utilized across various populations, with numerous translations accessible at no cost. It is regarded as a dependable and valid instrument and is widely acknowledged in research. A CES-D cut-off score of 16 appears to be suitable for most populations, mainly when the objective is to identify individuals with a heightened risk of major depressive disorder [44]. It may be necessary to slightly lower the CES-D cut-off to identify individuals with dysthymic disorder or minor depressive disorder.

- Hospital Anxiety and Depression Scale (HADS)

Description: This scale comprises 14 items, with 7 items measuring cognitive and emotional aspects of depression, specifically anhedonia, and the remaining 7 items focusing on cognitive and emotional aspects of anxiety. This assessment does not include items about physical or emotional disorders [34,42].

Practical application: The HADS tool can be ordered via the website at https://www.gl-assessment.co.uk/assessments/products/hospital-anxiety-depression-scale/ (accessed on 12 October 2023). The total score ranges from 0 to 42, and the scores for the Hospital Anxiety and Depression Scale-Depression (HADS-D) and Hospital Anxiety and Depression Scale-Anxiety (HADS-A) subscales range from 0 to 21. The cut-off intervals for the subscales are as follows: 0–7 = normal, 8–10 = mild, 11–15 = moderate, and ≥16 = severe [46]. High scores show greater severity.

Psychometric properties: For this scale, Cronbach’s score ranges from 0.82 to 0.90 for HADS-D and from 0.78 to 0.93 for HADS-A. Various cut-off points from 8 to 11 have been established [46]. The sensitivity and specificity for both HADS-A and HADS-D are approximately 0.80 [47].

Strengths and limitations: This intervention is characterized by its high level of time efficiency, extensive utilization across diverse demographics, and the availability of several translated versions. Although the HADS is a concise tool, it effectively assesses potential symptoms of anxiety and depression, exhibiting similarities to more extensive clinical assessments. Due to the absence of an item about suicidal ideation, the Hospital Anxiety and Depression Scale (HADS) primarily addresses less severe manifestations of these conditions.

- Patient Health Questionnaire-9 (PHQ-9)

Description: It consists of nine items aligned with the Diagnostic and Statistical Manual of Mental Disorders (DSM) and an additional item addressing impairment in work, daily functioning, or interpersonal relationships due to major depression. Patients indicate their condition for each item based on their experiences in the two weeks prior to the assessment [35]. The subsequent variants of the PHQ-9, the PHQ-2 and the PHQ-8, consist of two items and eight items, respectively. The PHQ-2 encompasses the two initial items of the PHQ-9, which assess depressive mood and loss of interest. It has demonstrated favorable psychometric properties, indicating its promising utility as a screening tool for depression [48]. The PHQ-8 excludes a question regarding thoughts of death or self-harm. It is applied in epidemiological research, particularly when examining infrequent responses or depression as secondary variables [49].

Practical application: The PHQ-9 has been translated and adapted to numerous languages and is accessible for free at https://www.phqscreeners.com/ (accessed on 12 October 2023). Various administration methods are available, such as self-reporting using pencil and paper, computer touch screen, direct interview, or telephone interview. Typically, the administration process lasts approximately 3 min and does not require specialized training in either administration or scoring. A 4-point scale indicates the degree of severity; items are rated from 0 (not at all) to 3 (nearly daily). The total score ranges between 0 and 27. The following cut-off points for measuring the severity of depression were proposed by the authors: 1–4, no depression; 5–9, mild depression; 10–14, moderate depression; 15–19, moderately severe depression; and 20–27, severe depression. A score of 10 is the recommended cut-off indicative of a diagnosis of major depression [35]. The authors suggest that to diagnose major depressive disorder (MDD), at least five of the nine symptom criteria should be present for over half the days in the past two weeks. Additionally, one of these symptoms should be a depressed mood or anhedonia, or the individual should have thoughts of self-harm or death. If two, three, or four of the symptom criteria were present at least half of the days in the past two weeks, along with a depressed mood or anhedonia, other depressive disorders should be considered [35].

Psychometric properties: The PHQ-9 shows high internal consistency, as evidenced by Cronbach’s alpha coefficients of 0.89 and 0.86. Based on interviews with mental health professionals, it was found that a PHQ-9 score of 10 or higher accurately detected major depression with a sensitivity of 0.88 and a specificity of 0.88. These findings were consistent across primary care and obstetrics/gynecology samples [35]. It is recommended to use a decrease of five points in the PHQ-9 score to measure a substantial treatment response or a reduction in depression [50].

Strengths and limitations: The PHQ-9 is a good screening and case-finding tool with moderate-to-good psychometric properties that is widely used in many populations. Additionally, it exhibits sensitivity towards treatment outcomes and can be applied for both the diagnosis of depressive disorders and the assessment of depression severity. Several systematic reviews and meta-analyses showed high specificity but moderate sensitivity, with uncertainty about the appropriate cut-off score to be utilized, as this decision depends on the specific attributes of the population and the context in which the assessment is conducted [46,51,52].

- Beck Depression Inventory (BDI-II)

Description: The BDI is a widely used self-reported rating scale for identifying and quantifying the psychopathological severity of depressive conditions [53,54]. There are four versions of the BDI: the original BDI, first published in 1961 [42] and later revised in 1978 as the BDI-IA; the BDI-II, published in 1996 [36]; and the BDI for Primary Care (BDI-PC), known as the BDI-FS [55,56]. The BDI-II’s content validity seems to be sufficient but is less expansive than that of the previous iteration. Six of the nine criteria for DSM-based depression were included in the BDI-I; however, the BDI-II showed an amended specificity to denote DSM-based depression. As a result, the BDI-II’s ability to detect depression in its broadest sense was improved [36].

Practical application: To purchase the BDI-II and BDI-FS manuals and instruments, access the online website: www.pearsonassesments.com (accessed on 12 October 2023). The standardized method for administering the instruments, whether in group or individual settings, is through paper-and-pencil self-application. Additionally, it is acceptable for the administrator to verbally instruct the participant in cases where their reading skills are limited, such as for participants with poor eyesight, low levels of education, or difficulties with concentration. The BDI-II has a relatively low burden in terms of its application, typically requiring a time commitment of approximately 5–10 min.

The BDI-II instrument consists of 21 items measuring cognitive, affective, somatic, and vegetative symptoms of depression corresponding to criteria from the DMS-IV. Each item is scored from 0 (not at all) to 3, with a higher score denoting more severe depression [36,37]. The BDI-FS includes 13 items that exclude some somatic criteria and refer to cognitive and affective symptoms of depression [37]. The score ranges in Table 2 have been suggested to quantify the severity of depression [53,56].

Psychometric properties: The internal consistency of the BDI-II demonstrates strong reliability, with values of 0.93 for university students and 0.92 for psychiatric patients. Also, the internal consistency of the BDI-II was strong across several language translations, as shown by alpha values ranging from 0.73 to 0.96 [37,42].

Strengths and limitations: Among the existing tools for measuring depression, the BDI-II is concise and user-friendly, with broad content coverage for depressive symptoms, good reliability across languages, and easy symptom screening and reassessment. The BDI-II shares similar limitations with other self-administered questionnaires since respondents can manipulate their scores by inflating, minimizing, or fabricating their responses. Moreover, it aligns with the DSM-IV, but not the DSM-5, and needs a purchased license.

- Zung Self-Rating Depression Scale (SDS)

Description: The Zung Self-Rating Depression Scale is a short self-administered survey to quantify the depressed status of a patient. This scale consists of 20 items that assess four prevalent aspects of depression, namely its pervasive impact, physiological manifestations, additional problems, and impaired psychomotor behaviors [38].

Practical application: The SDS is available in the original article [38] and online at https://integrationacademy.ahrq.gov/sites/default/files/202007/Zung_Self_Rating_Depression_Scale.pdf/ (accessed on 12 October 2023). Item responses are assigned numerical ratings ranging from 1 to 4, where higher scores indicate a greater frequency of symptoms. The intensity of depressive symptoms is represented by the standardized score, which is calculated by multiplying the total of the raw item scores of the 20 items by a factor of 1.25. Within this range, scores falling between 53 and 62 indicate mild depression, scores between 63 and 72 indicate moderate depression, and scores between 72 and 100 indicate severe depression. The established clinical threshold for depression is 53 [38].

Psychometric properties: This tool demonstrates satisfactory internal consistency, as indicated by a split-half reliability coefficient of 0.73. Deforge and Sobal found an alpha coefficient of 0.68 [57]. However, other researchers have reported higher values of 0.87 [58]. For the 50-point cut-off applied by many researchers, sensitivity is 78.9% and specificity is 83.7% [58].

Strengths and limitations: This scale is generally considered a reliable instrument for assessing the severity of depression in primary care. The variability of the cut-off values used in the literature is used to maximize the benefit of testing, and all factors must be considered. A cut-off point ≥ 50 is recommended for clinical significance [38,58].

- Generalized Anxiety Disorder-7 (GAD-7)

Description: It is a recently developed, easy-to-use questionnaire with strong psychometric properties. It involves seven items based on the Diagnostic and Statistical Manual of Mental Disorders-IV (DSM-IV) criteria, and it identifies cases of generalized anxiety [59]. There were some recommendations to condense the questionnaire further by utilizing just the two initial inquiries of the GAD-7, which pertain to the primary manifestations of generalized anxiety disorder. Consequently, the GAD-2 was developed, and it serves as a condensed iteration of the GAD-7 that incorporates the two initial questions, which represent the primary manifestations of anxiety [39].

Practical application: The questionnaire is available in many languages at https://www.phqscreeners.com/ (accessed on 15 October 2023) and takes 5 min to complete. Each question is scored on a scale ranging from 0 to 3, and they examine the frequency of seven distinct anxiety symptoms experienced by the patient in the last two weeks. The patient’s response options include “not at all”, “several days”, “more than half the days”, and “nearly daily”, which are assigned scores of 0, 1, 2, and 3, respectively. Mild anxiety is defined as a score of 5, while a score of 10 indicates moderate anxiety. Severe anxiety is identified by a score of 15 [59].

Psychometric properties: A cut-off point ≥ 10 demonstrates a sensitivity of 89% and a specificity of 82% for generalized anxiety disorder. The GAD-7 scale has an excellent internal consistency of 0.92 and good test–retest reliability (intraclass correlation = 0.83) [59].

Strengths and limitations: The GAD-2 and GAD-7 questionnaires are efficient with regards to the time of administration, which boosts their time-effectiveness. Both questionnaires demonstrate high sensitivity and specificity in accurately diagnosing the prevalent anxiety disorders commonly observed in primary care settings. The GAD-7 instrument offers potential diagnostic indications that require additional investigation for confirmation.

- State-Trait Anxiety Inventory (STAI)

Description: The STAI is a validated 40-item self-report tool that separately measures the temporary condition of state anxiety and the more general and long-standing quality of trait anxiety [60].

Practical application: This inventory can be purchased at https://www.mindgarden.com/state-trait-anxiety-inventory-for-adults/771-staiad-license-to-administer.html (accessed on 15 October 2023). The State-Trait Anxiety Inventory assesses the present level of anxiety. The STAI-State gathers responses on a scale from 0 to 3, ranging from “not at all” to “very much so” for several elements such as concern, apprehension, uneasiness, tension, and autonomic nervous system activation/arousal. These responses reflect the individual’s current emotional state. The STAI-Trait instrument evaluates enduring characteristics related to “anxiety proneness” or the regularity of experiencing anxiety (e.g., overall levels of self-assurance, tranquility, or assurance). The STAI-Trait questionnaire utilizes a Likert scale ranging from “almost never” to “almost always” with numerical representations from 0 to 3. Scores range from 20 to 80, and higher scores indicate more severe anxiety [40].

Psychometric properties: Internal consistency coefficients for this scale have ranged from 0.86 to 0.95 [60].

Strengths and limitations: This scale is reliable for assessing anxiety, including its state form and trait form, and is easy to apply. This questionnaire can be used in research after obtaining the author’s permission and purchasing the form.

- Beck Anxiety Inventory (BAI)

Description: The BAI is a diagnostic instrument utilized to assess the intensity of anxiety symptoms experienced by individuals, independent of any potentially overlapping symptoms of depression or other disorders. Many items focus on physiological or psychosomatic symptoms rather than cognitive symptoms [41].

Practical application: This scale is copyrighted. It is available for purchase from Psychological Corporation, 555 Academic Court, San Antonio, TX, 78204-2498, USA, and can be purchased online at www.pearsonassesments.com (accessed on 15 October 2023). This questionnaire consists of 21 items, either self-administered or verbally delivered by a professional, and it takes 5–10 min to complete. The patient rates how much he or she has been bothered by each symptom over the past week on a 4-point scale from 0 (not at all) to 3 (severely). A score of 0 to 7 indicates minimal anxiety, 8 to 15 indicates mild anxiety, 16 to 25 indicates moderate anxiety, and 30 to 63 indicates severe anxiety.

Psychometric properties: Based on a meta-analysis that involved a total of 117 studies, it was shown that the BAI demonstrated a high level of internal consistency in both clinical (0.91) and non-clinical (0.91) samples. Additionally, the BAI exhibited satisfactory test–retest reliability in clinical (0.66) and non-clinical (0.65) populations [41,61].

Strengths and limitations: The BAI discriminates anxious diagnostic groups (panic disorder and generalized anxiety disorder) from non-anxious diagnostic groups like major depression and dysthymic disorder. There is an issue concerning the efficacy of the BAI’s clinical utility as an anxiety screening tool and a measurement of severity in primary care settings. While the BAI was not initially designed for diagnostic purposes, assessing its diagnostic reliability and score distribution in a sample is crucial before employing it for anxiety screening, monitoring symptom changes, or measuring outcomes based on severity.

Table 3 evaluates the strengths and limitations of each scale and the study populations in which the questionnaires have been validated.

In Table 4, we summarize the characteristics of the clinical studies identifying depression and anxiety in OSA patients using the most relevant self-reported scales.

## 4. Discussion

The prevalence of depression across the included studies ranged from 14.4% [93] to 88.3% [101], and that of anxiety ranged from 15.9% [93] to 62.2% [88]. A review showed that prevalence figures fluctuated considerably for both depression (7–63%) and anxiety (11–70%) in patients with OSA [107]. Moreover, Garbarino et al. found similar results for depression (2.9% to 78%) and anxiety (2.9% to 70%) [10]. Regarding results, most of the research concentrated on depression or both anxiety and depression; we encourage clinicians to assess both because, most of the time, they coexist in OSA patients.

The association of OSA with mental health issues, especially depression and anxiety, is receiving increased attention due to its impact on everyday quality of life. In recent decades, a great joint effort has been dedicated to building assessment tools to screen for depression and anxiety in large community studies. Self-reported scales are easy to administer, cost-effective, do not require extensive training programs, and are less influenced by interviewers’ expectations. Nevertheless, the effectiveness of these surveys relies on the respondent’s willingness to participate and their comprehension of the questions. The information gathered from these questionnaires can guide discussions with patients, enhance patient–provider communication, and inform decision making regarding appropriate interventions, such as psychotherapy or pharmacotherapy.

There are currently multiple tools that can be used to screen for depression and anxiety and measure their severity in OSA patients, and these scales have been summarized in this narrative review. The selection of the most appropriate instrument for assessing and rating depression and anxiety should be guided by the scale’s appropriateness for the study’s objective, availability, psychometric attributes, and previous experience in similar populations.

For this study, the most common questionnaire used to screen for depression was the Beck Depression Inventory (BDI, BDI-FS, and BDI-II), which was used in 11 studies (45.8%). The Hospital Anxiety and Depression Scale (HADS-D) was used in five studies (20.8%), the Patient Health Questionnaire-9 (PHQ-9) and the Zung Self-Rating Depression Scale (SDS) were used in three studies each (12.5%), and the Center for Epidemiologic Studies Depression Scale (CES-D) was used in two studies (8.3%). For anxiety, the Hospital Anxiety and Depression Scale (HADS-A) was used in five studies (20.8%), the Generalized Anxiety Disorder scale (GAD-7) was used in two studies (8.3%), and the State-Trait Anxiety Inventory (STAI) and Beck Anxiety Inventory (BAI) were used in one study each (4.1%).

In 2015, Pettersson et al. conducted a comprehensive study of 20 diagnostic tools for depression. The PHQ-9 self-report questionnaire, with a cut-off point of 10, met the minimum criteria for specificity, with a specificity of 0.88 and a sensitivity of 0.78. The Hospital Anxiety and Depression Scale, when using a cut-off point of 7, demonstrated insufficient sensitivity (0.70) for practical application in clinical settings. Similarly, the BDI-II, with a cut-off point of 14, shows inadequate specificity (0.72) [108].

In another study conducted by Yuan et al. in 2019, the PHQ-9 and HADS-D screening scales were compared for diagnosing major depression in 782 Chinese patients with acute coronary syndrome. The diagnostic accuracy of the scales was similar, with AUC values of 0.84 and 0.81, respectively. The standard cut-off point of 10 was determined to be the optimal point for this study. The scales exhibited similar specificity values of 0.85 and 0.86, while the PHQ-9 demonstrated higher sensitivity at 0.87 compared to 0.76 for the HADS-D [109].

Wang et al. showed in a systematic review that the BDI-II had convergent validity with other scales like the CES-D, the HASD-D, the Hamilton Depression Rating Scale, and the Edinburg Postnatal Depression Scale, ranging from 0.62 to 0.81 [110].

A recent systematic review from 2020 that evaluated validated screening tools for anxiety disorders and post-traumatic stress disorder in low- to middle-income countries showed that the most common tool used to screen for anxiety disorder was the Kesller-10, followed by the GAD-7 [111].

The results of our study indicated that the Beck Depression Inventory (BDI, BDI-FS, and BDI-II) and the Hospital Anxiety and Depression Scale (HADS-D) were used the most to identify depression in OSA patients. The major limitation of these scales is that they are not free to use, unlike the PHQ-9. Additionally, the PHQ-9 has an advantage over these scales, as it includes a suicidal ideation item, which highlights the need for patients to seek help from specialized professionals. Regarding anxiety, we found that the HADS-A, followed by the GAD-7 questionnaire, was the most used, as in previous results. The GAD-7 is free to use, and compared with the STAI and BAI, it has been validated in many study populations.

A limitation of this study is the fact that the majority of the research evaluated depression or both depression and anxiety. Future research needs to be directed towards evaluating anxiety alone in OSA patients. Another limitation is that potential cases of depression and anxiety are identified by self-reported screening measures, in which the scores can be easily exaggerated, minimized, or even falsified by the respondents. Individuals with additional medical conditions tend to exaggerate physical symptoms, such as tiredness and alterations in sleep patterns, which can increase questionnaire scores and lead to overestimations of the presence of symptoms. Several gaps need to be addressed to adequately research the evaluation of depression and anxiety related to comorbidities, socioeconomic level, and medication intake in populations with obstructive sleep apnea. In addition, more research needs to explore the underlying mechanisms linking OSA with mental health comorbidities. This will allow for a deeper understanding of the shared pathophysiology and potential treatment targets.

## 5. Conclusions

Clinicians should know the advantages and challenges of using self-reported scales for the assessment of mental health comorbidities in OSA patients and the importance of routine screening due to the high prevalence of depression and anxiety. Results should be interpreted with clinical evaluations and other objective measures to ensure accurate diagnosis and treatment planning. Integrating mental health assessments into routine clinical care for individuals with OSA can improve the detection and management of these comorbidities, enhancing overall treatment outcomes and quality of life.

## Figures and Tables

**Table 1:** Screening and rating tools for depression and anxiety.

Scales	Overview	Number of Items	Scoring	Cut-Off	Time Frame	Administration
CES-D [33]	Screening for depression	20	Responses use a 4-point scale (0—rarely or none of the time (less than 1 day), 1—some or a little of the time (1–2 days), 2—occasionally or a moderate amount of time (3–4 days), and 3—most or all of the time (5–7 days)), with a total score ranging from 0 to 60.	≥16	Past week	10 min
HADS-A HADS-D [34]	Screening for depression and anxiety	14	It comprises 14 items, 7 for anxiety and 7 for depression, and is rated from 0 to 3, according to how the respondent has felt during the past week. Total scores range from 0 to 42 and subscales range from 0 to 21.	≥8	Past week	2–5 min
PHQ-9 [35]	Screening for depression	9	A 4-point scale indicates the degree of severity; items are rated from 0 (not at all) to 3 (nearly daily). Total score ranges between 0 and 27.	≥10	Past 2 weeks	3–5 min
BDI-II [36,37]	Rating depression	21	It questions how respondents have felt recently. It is rated using a scale of increasing ordinal severity, ranging from 0 to 3. The total score varies from 0 to 63.	≥11	Not established	5–10 min
SDS [38]	Rating depression	20	The standardized score is calculated by multiplying the total of the raw item scores of the 20 items by a factor of 1.25. They are scored from 0 (some of the time) to 4 (most of the time). The range is 0–100, where higher scores indicate more severe depression.	≥53	Past several days	5–10 min
GAD-7 [39]	Screening for anxiety	7	The patient’s response options include “not at all,” “several days,” “more than half the days,” and “nearly daily,” which are assigned scores of 0, 1, 2, and 3, respectively. Mild anxiety is defined as a score of 5, while a score of 10 indicates moderate anxiety.	≥10	Past 2 weeks	3–5 min
STAI [40]	Rating anxiety	40	STAI-State responses use a scale of 0 to 3, ranging from “not at all” to “very much so”. These responses reflect the individual’s current emotional state. STAI-Trait responses range from 0 (almost never) to 3 (almost always). Scores range from 20 to 80, and higher scores indicate more severe anxiety.	Not established	State anxiety Trait anxiety	10–20 min
BAI [41]	Rating anxiety	21	The total score varies from 0 to 63. A score of 0 to 7 indicates minimal anxiety, 8 to 15 indicates mild anxiety, 16 to 25 indicates moderate anxiety, and 30 to 63 indicates severe anxiety.	≥16	Past week	10 min

CES-D, The Center for Epidemiologic Studies Depression Scale; PHQ-9, The Patient Health Questionnaire-9; HADS-A and HADS-D, The Hospital Anxiety and Depression Scale; BDI-II, Beck Depression Inventory; SDS, The Zung Self-Rating Depression Scale; STAI, State-Trait Anxiety Inventory; BAI, Beck Anxiety Inventory; GAD-7, Generalized Anxiety Disorder-7.

**Table 2:** Score range suggestions for interpretation.

Score Range	BDI-II	BDI-FS	BDI-IA
No/minimal depression	0–13	0–3	0–9
Mild depression	14–19	4–8	10–16
Moderate depression	20–28	9–12	17–29
Severe depression	29–63	13–21	30–63

BDI-II: Beck Depression Inventory, version II; BDI-FS: Beck Depression Inventory, Fast Screen in Medical Patients; BDI-IA: Beck Depression Inventory, first revision.

**Table 3:** The strengths and weaknesses of the tools and their validity in various populations.

Tool	Strengths	Weaknesses	Validity in Study Populations
CES-D [33]	Alpha: 0.90 Sensitivity: 74.6% Specificity: 73.4% Free to use	A cut-off of 20 may be better than the value of 16 which is typically recommended [44].	General population [33], primary care [62], oncology [63], diabetes [64], systemic sclerosis [65], stroke [66]
HADS [34]	Alpha: 0.83 Sensitivity: 80% Specificity: 80%	A license must be purchased for use. Absence of suicidal ideation item. Variation of cut-off points in studies.	General population, primary care [47], COPD [67], oncology [68], multiple sclerosis [69], Parkinson’s disease [70]
PHQ-9 [35]	Alpha: 0.86 Sensitivity: 88% Specificity: 88% Free to use Suicidal ideation item	A cut-off ≥ 10 could overestimate the symptoms [18].	Diabetes [64], systemic sclerosis [65], rheumatological disorders [71], oncology [72], immunodeficiency disorder [73]
BDI [36,37]	Alpha: 0.92–0.93 Test–retest reliability: 0.73–0.96 Sensitivity: >70%	A license must be purchased for use. Aligns with DSM-IV but not DSM-5. Usually used as the first application without having a previous depression diagnosis.	General population [37], diabetes [64], oncology [74], Parkinson’s disease [75]
SDS [38]	Alpha: 0.92–0.93 Test–retest reliability: 0.73–0.96 Sensitivity: >70%	A license must be purchased for use. The large number of somatic items is likely to inflate depression rates. Requires more evidence of validity in various populations.	Older adults [76]
GAD-7 [39]	Alpha: 0.83 Sensitivity: 89% Specificity: 82% Free to use	Scores can be easily exaggerated.	General population [77], multiple sclerosis [69], oncology [78], epilepsy [79], COPD [80]
STAI [60]	Alpha: 0.86–0.95 Test–retest reliability: 0.65–0.75 Sensitivity: 78.3% Specificity: 71.2%	A license must be purchased for use. Based on DSM-IV criteria. More items.	Urologic diseases [81], oncology [82], multiple sclerosis [69]
BAI [41,83]	Alpha: 0.91 Test–retest reliability: 0.58–0.66 Sensitivity: >70%	A license must be purchased for use. Aligns with DSM-IV but not DSM-5. Requires more evidence of validity in various populations.	Multiple sclerosis [69]

CES-D, The Center for Epidemiologic Studies Depression Scale; PHQ-9, The Patient Health Questionnaire-9; HADS-A and HADS-D, The Hospital Anxiety and Depression Scale; BDI-II, Beck Depression Inventory; SDS, The Zung Self-Rating Depression Scale; STAI, State-Trait Anxiety Inventory; BAI, Beck Anxiety Inventory; GAD-7, Generalized Anxiety Disorder-7; COPD, chronic obstructive pulmonary disease; DSM-IV and DSM-5, Diagnostic and Statistical Manual of Mental Disorders.

**Table 4:** Screening tools, the prevalence of depression and anxiety symptoms in OSA, and cut-offs.

Study ID	Tool for Evaluation	Study Design	Sample Size (Participants)	Mean Age (SD) % Females	Prevalence of Mental Disorders	Cut-Off and Severity
Bardwell et al., 2003 [84]	CES-D	Cross-sectional	60	49.1 (7.5) 15.6%	In total, 33.3% of OSA patients presented depressive symptoms.	≥16
Diamanti et al., 2013 [85]	CES-D	Prospective observational	41	51.9 (10.5) 14.6%	In total, 53.6% of OSA patients presented depressive symptoms.	≥16
Daabis et al., 2012 [86]	HADS-A HADS-D	Case–control	102	48.8 (11.73) 17%	In total, 33% of OSA patients presented anxiety symptoms, and 51% presented depressive symptoms.	≥11
Surani et al., 2013 [87]	HADS-A HADS-D	Cross-sectional	51	No data	In total, 52.9% of OSA patients presented anxiety symptoms, and 39.2% presented depressive symptoms.	≥10
Akberzie et al., 2018 [88]	HADS-A HADS-D	Cross-sectional	45	47 (No data) 64%	In total, 62.2% of OSA patients presented anxiety symptoms, and 64.4% presented depressive symptoms.	≥8
Lundetræ et al., 2021 [89]	HADS-A HADS-D	Prospective observational	468	55.5 (12) 28.8%	In total, 26.3% of OSA patients presented anxiety symptoms, and 17.5% presented depressive symptoms.	≥8
Walker et al., 2021 [90]	HADS-A HADS-D	Prospective observational	108	56 (12.8) 27.8%	In total, 17.6% of OSA patients presented anxiety symptoms, and 37% presented depressive symptoms.	HADS-A ≥ 8 HADS-D ≥ 11
Edwards et al., 2015 [91]	PHQ-9	Prospective observational	293	52 (No data) 38.3%	In total, 72.6% of OSA patients presented depressive symptoms.	≥10
Velescu et al., 2022 [92]	PHQ-9 GAD-7	Prospective observational	99	56 (10.92) 32.67%	In total, 48.5% of OSA patients presented depressive symptoms, and 27.3% presented anxiety symptoms.	≥10
Lee et al., 2023 [93]	PHQ-9 GAD-7	Cross-sectional	1390	50 (12.4) 19.6%	In total, 15.9% of OSA patients presented anxiety symptoms, and 14.4% presented depressive symptoms.	PHQ-9 ≥ 10 GAD-7 ≥ 8
McCall et al., 2006 [94]	BDI	Cross-sectional	121	51.7 (14.1) 24%	In total, 44.6% of OSA patients presented depressive symptoms.	≥10
Lee, W et al., 2015 [95]	BDI	Cross-sectional	302	48.4 (11.3) Only men	In total, 39% of OSA patients presented depressive symptoms.	≥10
Yosunkaya et al., 2016 [96]	BDI	Cross-sectional	200	45.5 (9.9) 12.5%	In total, 16.4% of OSA patients presented moderate depressive symptoms.	≥17
Karamanli et al., 2016 [97]	BDI	Case–control	96	51.4 (1.3) 41.6%	In total, 59,7% of OSA patients presented depressive symptoms, and 25% had moderate to severe depression.	mild (10–15), moderate (16–23), and severe (24–63)
Schwartz et al., 2007 [98]	BDI-FS	Prospective observational	50	53 (11.3) 22%	In total, 33% of OSA patients presented depressive symptoms.	≥4
Aloia et al., 2005 [99]	BDI-II	Cross-sectional	93	52.2 (11.1) 34.4%	In total, 33.3% of OSA patients presented depressive symptoms.	≥14
Cross et al., 2008 [25]	BDI-II	Case–control	101	47.6 (11) No data	In total, 33% of OSA patients presented elevated depressive symptoms (BDI-II ≥ 12).	≥12 symptomatic ≤10 asymptomatic
Ishman et al., 2014 [100]	BDI-II	Prospective observational	104	46.8 (9.1) 24.5%	In total, 27.3% of OSA patients presented depressive symptoms.	≥12
Chirinos et al., 2017 [101]	BDI-II	Cross-sectional	181	48.9 (11.2) No data	88.3% minimal 8.9% mild 2.2% moderate 0.6% severe depression	Minimal: 0–13 Mild: 14–19 Moderate: 20–28 Severe: 29–63
Yamatoto et al., 2000 [102]	SDS	Prospective observational	47	49.5 (10.8) No data	In total, 63.4% of OSA patients presented depressive symptoms.	≥41
Dai et al., 2016 [103]	SDS	Cross-sectional	1327	47 (No data) 19.3%	In total, 47.4% of OSA patients presented depressive symptoms.	≥53
Balcan et al., 2019 [104]	SDS	Cross-sectional	493	63.9 (8.6) 16.8%	In total, 29.3% of OSA patients presented depressive symptoms.	≥50
Lee et al., 2015 [105]	STAI BDI	Cross-sectional	655	49.8 (11.70) 13.1%	In total, 48.4% of OSA patients presented anxiety symptoms, and 46.4% presented depressive symptoms.	STAI ≥ 40 BDI ≥ 10
Rezaeitalab et al., 2014 [106]	BAI BDI	Cross-sectional	178	50.3 (No data) 14.4%	In total, 53.9% of OSA patients presented anxiety symptoms, and 46.1% presented depressive symptoms.	BAI ≥ 8 BDI ≥ 10

CES-D, The Center for Epidemiologic Studies Depression Scale; PHQ-9, The Patient Health Questionnaire-9; HADS-A and HADS-D, The Hospital Anxiety and Depression Scale; BDI-II, Beck Depression Inventory; SDS, The Zung Self-Rating Depression Scale; STAI, State-Trait Anxiety Inventory; BAI, Beck Anxiety Inventory; GAD-7, Generalized Anxiety Disorder-7; No data, data not available in the article.

## Data Availability

The data used in this study are available from the corresponding authors upon request.
